# Comprehensive AI-assisted tool for ankylosing spondylitis based on multicenter research outperforms human experts

**DOI:** 10.3389/fpubh.2023.1063633

**Published:** 2023-02-09

**Authors:** Hao Li, Xiang Tao, Tuo Liang, Jie Jiang, Jichong Zhu, Shaofeng Wu, Liyi Chen, Zide Zhang, Chenxing Zhou, Xuhua Sun, Shengsheng Huang, Jiarui Chen, Tianyou Chen, Zhen Ye, Wuhua Chen, Hao Guo, Yuanlin Yao, Shian Liao, Chaojie Yu, Binguang Fan, Yihong Liu, Chunai Lu, Junnan Hu, Qinghong Xie, Xiao Wei, Cairen Fang, Huijiang Liu, Chengqian Huang, Shixin Pan, Xinli Zhan, Chong Liu

**Affiliations:** ^1^The First Affiliated Hospital of Guangxi Medical University, Nanning, Guangxi, China; ^2^Guangxi Medical University, Nanning, Guangxi, China; ^3^Orthopaedics of The First People's Hospital of Nanning, Nanning, Guangxi, China; ^4^Orthopaedics of People's Hospital of Baise, Baise, Guangxi, China; ^5^Orthopaedics of Wuzhou Red Cross Hospital, Wuzhou, Guangxi, China

**Keywords:** artificial intelligence, deep learning, machine learning, ankylosing spondylitis, pelvic radiograph

## Abstract

**Introduction:**

The diagnosis and treatment of ankylosing spondylitis (AS) is a difficult task, especially in less developed countries without access to experts. To address this issue, a comprehensive artificial intelligence (AI) tool was created to help diagnose and predict the course of AS.

**Methods:**

In this retrospective study, a dataset of 5389 pelvic radiographs (PXRs) from patients treated at a single medical center between March 2014 and April 2022 was used to create an ensemble deep learning (DL) model for diagnosing AS. The model was then tested on an additional 583 images from three other medical centers, and its performance was evaluated using the area under the receiver operating characteristic curve analysis, accuracy, precision, recall, and F1 scores. Furthermore, clinical prediction models for identifying high-risk patients and triaging patients were developed and validated using clinical data from 356 patients.

**Results:**

The ensemble DL model demonstrated impressive performance in a multicenter external test set, with precision, recall, and area under the receiver operating characteristic curve values of 0.90, 0.89, and 0.96, respectively. This performance surpassed that of human experts, and the model also significantly improved the experts' diagnostic accuracy. Furthermore, the model's diagnosis results based on smartphone-captured images were comparable to those of human experts. Additionally, a clinical prediction model was established that accurately categorizes patients with AS into high-and low-risk groups with distinct clinical trajectories. This provides a strong foundation for individualized care.

**Discussion:**

In this study, an exceptionally comprehensive AI tool was developed for the diagnosis and management of AS in complex clinical scenarios, especially in underdeveloped or rural areas that lack access to experts. This tool is highly beneficial in providing an efficient and effective system of diagnosis and management.

## Introduction

The exact cause of ankylosing spondylitis (AS), a chronic inflammatory illness, remains unknown (1). AS is characterized by inflammation and stiffness, mainly at the cartilage–boneinterface (2). Risk factors for the condition include heredity and the environment, and it is more prevalent in younger males. AS is thought to be an immune-mediated illness accompanied by inflammatory cell infiltration (1, 3). AS is accompanied by several comorbidities, chronic pain, functional impairment, and resource consumption which places an economic burden on the nation and society (4–6). Early, consistent therapy may successfully slow the disease progression. Many medications have a positive effect and can ease patients' symptoms and enhance their quality of life (7, 8). However, not every patient with AS receives prompt diagnosis and care. Misdiagnosis and missed diagnosis frequently result in delayed diagnosis, which prevents patients from receiving treatment at the most effective time (9). Due to serious lesions on the spine or hip joint, many patients with AS ultimately lose the capacity to care for themselves and surgery remains the only option. The quality of life for patients with AS can be improved by surgery, thanks to the highly developed surgical technologies currently available (10, 11). However, compared to patients with other degenerative illnesses, patients with AS are at a significantly higher risk for surgery and major post-operative complications (12–16). For early diagnosis, magnetic resonance imaging (MRI) can help identify the early lesions of sacroiliitis, specifically bone marrow edema (3). Deep learning (DL) techniques have been employed to create models that can increase the diagnostic effectiveness of MRI (17, 18). These models perform well during validation; their area under curve (AUC) values are higher than 0.9. Inflammatory abnormalities in the sacroiliac joints can be detected early using MRI, and DL techniques can further boost diagnostic precision. For developed nations, this is feasible. However, this approach may not be feasible in some developing nations due to the lack of MRI equipment. Moreover, because MRI examinations are expensive, using them as the main screening tool would put an immense financial strain on patients. Patients with AS often tend to ignore the initial symptoms and seek medical assistance at the closest medical facilities, many of which are community hospitals without MRI technology. Pelvic radiograph (PXR) examinations are reasonably priced and can be used to detect early sacroiliitis lesions. PXR examinations can be performed by all medical facilities and are common in developing and impoverished countries. According to the modified New York criteria (3), AS can be identified if the bilateral sacroiliitis scores on the PXRs are higher than 2, or if the score on either side was higher than 3, along with some clinical symptoms. Patients with AS often seek medical assistance for clinical symptoms such as lower-back discomfort and limited lumbar spine motion; the diagnosis can be performed if the patient meets the imaging criteria. Therefore, by using PXR, a skilled radiologist, rheumatologist, or orthopedic surgeon can swiftly diagnose whether a patient has AS. This is a very effective technique that prevents needless medical resource wastage in addition to being affordable. However, AS is an uncommon condition compared to more widespread conditions such as lumbar disc herniation. Underdeveloped regions, in particular, lack sufficient AS diagnostic professionals. Although diagnosing AS is not challenging in institutions with experts, it is challenging in most primary hospitals to identify AS early and advise patients to seek treatment from a specialist. As a result, AS is often overlooked or misdiagnosed in developing nations, delaying diagnosis and preventing patients from receiving the best possible care (9).

Artificial intelligence (AI) systems can be employed to aid in diagnosis, particularly the high-performing DL models, which can greatly increase the accuracy of AS diagnosis in developing countries, especially in underdeveloped areas. Convolutional neural networks (CNNs) are typically used to analyze image data. The prediction accuracy of CNNs is higher than that of human experts in numerous medical datasets, including the identification of aberrant electrocardiograms (19), early diagnosis of biliary atresia (20), and detection of pelvic injuries (21). However, to date, no large-scale, multicenter investigation has been performed on the early detection of AS by using CNN. PXR examination is very common in both primary care settings and tertiary hospitals, and any high-quality AI model based on PXR would considerably improve diagnostic accuracy where primary hospitals lack expertise. However, many primary hospitals lack not only diagnostic skills but also the ability to assess the course of a disease and determine which patients require additional medical care. As a result, despite these institutions using diagnostic models to increase the accuracy of AS diagnosis, they fail to properly triage patients and achieve individualized care.

To close such gaps, in this study, we aimed to construct a comprehensive AI-assisted tool for AS diagnosis and AS patient clinical prediction. By using ensemble learning techniques, we established a model having the best performance in this field. The study results also demonstrate that combined diagnosis by human experts and the diagnostic model can increase the accuracy of diagnosis. In addition, we considered the fact that the hospital's X-ray equipments are typically not connected to the Internet, making it difficult to efficiently input images into the model. With the use of smartphone-captured images, our diagnostic model can perform accurate judgments, accomplishing in-the-moment intelligent diagnosis and further enhancing the practical applicability of the proposed model. To accomplish the most rational distribution of medical resources, we created an AI-assisted tool that not only significantly increases the diagnostic accuracy of radiologists but also enables early detection and shunting of high-risk groups, aids in realizing individualized therapy, and allows high-risk individuals to receive more medical attention.

## Overview

In this paper, we present the following sections: Results, Discussion, and Methods.

The Results Section covers: (1) basic information of the subjects and datasets included in the study; (2) performance of multiple ensemble deep learning diagnostic models in the internal validation set; (3) performance of above models in the multicenter external test set and selection of the optimal model; (4) testing of the optimal model using images taken by smartphones; (5) performance of clinical prediction models; (6) methods of model deployment.

The Discussion considers the following aspects: (1) the advantages of the AS deep learning diagnostic model; (2) the practicality of the model; (3) the interpretability of the model; (4) the training techniques of the model; (5) the excellent performance of the clinical prediction models and their clinical significance.

The Methods Section outlines the details of data collection, model training, and model evaluation criteria used in this study.

## Results

### Patient and dataset

For the ensembled DL diagnostic model, we utilized 5,389 PXRs from 5,014 patients from the First Affiliated Hospital of Guangxi Medical University as the training set and used 539 PXRs from three additional centers as the external test set. For demographic information, see Table 1.

**Table 1:** Characteristics of the study subjects of the DL model.

**Name**	**Levels**	**Testset (*N* = 539)**	**Trainset (*N* = 5,013)**	***p***
Gender	Female	169 (31.4%)	1,932 (38.5%)	0.001
	Male	370 (68.6%)	3,081 (61.5%)	
Age	Mean ± SD	29.7 ± 7.7	37.5 ± 12.0	< 0.001
Diagnosis	AS	238 (44.2%)	1,749 (34.9%)	< 0.001
	Non-AS	301 (55.8%)	3,264 (65.1%)	

In addition, we created Bath Ankylosing Spondylitis Functional Index (BASFI) and Bath Ankylosing Spondylitis Disease Activity Index (BASDAI) prediction models by using machine learning (ML) after collecting comprehensive clinical and imaging data of 356 patients with AS from the First Affiliated Hospital of Guangxi Medical University. The training set consisted of 284 patients, and the external test set included 72 patients. Data from the training set and external test set are described in Table 2.

**Table 2:** Characteristics of AS patients and comparison between trainset and testset.

**Name**	**Levels**	**Testset (*N* = 72)**	**Trainset (*N* = 284)**	***p***
Surgery	No	56 (77.8%)	213 (75%)	0.737
	Yes	16 (22.2%)	71 (25%)	
BASFI	Mean ± SD	2.7 ± 2.1	2.7 ± 2.1	0.867
BASDAI	Mean ± SD	1.9 ± 1.5	1.9 ± 1.4	0.826
Gender	Female	12 (16.7%)	42 (14.8%)	0.831
	Male	60 (83.3%)	242 (85.2%)	
Age (year)	< 34	36 (50%)	136 (47.9%)	0.851
	≥34	36 (50%)	148 (52.1%)	
BMI (kg/m^2^)	Mean ± SD	21.7 ± 2.8	22.1 ± 3.0	0.301
Occupation	Manual	17 (23.6%)	71 (25%)	0.927
	Non-manual	55 (76.4%)	213 (75%)	
Smoking	No	46 (63.9%)	175 (61.6%)	0.827
	Yes	26 (36.1%)	109 (38.4%)	
Drinking	No	58 (80.6%)	214 (75.4%)	0.439
	Yes	14 (19.4%)	70 (24.6%)	
Exercise	No	64 (88.9%)	247 (87%)	0.811
	Yes	8 (11.1%)	37 (13%)	
Onset (year)	< 20	12 (16.7%)	87 (30.6%)	0.027
	≥20	60 (83.3%)	197 (69.4%)	
Delay (year)	< 7	57 (79.2%)	212 (74.6%)	0.520
	≥7	15 (20.8%)	72 (25.4%)	
Duration (year)	< 10	53 (73.6%)	183 (64.4%)	0.183
	≥10	19 (26.4%)	101 (35.6%)	
Family history	No	62 (86.1%)	245 (86.3%)	1.000
	Yes	10 (13.9%)	39 (13.7%)	
Night pain	No	37 (51.4%)	135 (47.5%)	0.651
	Yes	35 (48.6%)	149 (52.5%)	
PGA	>3	18 (25%)	79 (27.8%)	0.740
	≤ 3	54 (75%)	205 (72.2%)	
Lumbar mobility	Limited	60 (83.3%)	245 (86.3%)	0.655
	Normal	12 (16.7%)	39 (13.7%)	
Cervical mobility	Limited	14 (19.4%)	39 (13.7%)	0.303
	Normal	58 (80.6%)	245 (86.3%)	
Fatigue	No	15 (20.8%)	52 (18.3%)	0.749
	Yes	57 (79.2%)	232 (81.7%)	
Spinal pain	Multiple locations	15 (20.8%)	57 (20.1%)	1.000
	Single location	57 (79.2%)	227 (79.9%)	
Hip involvement	No	29 (40.3%)	143 (50.4%)	0.163
	Yes	43 (59.7%)	141 (49.6%)	
Peripheral arthritis	No	66 (91.7%)	250 (88%)	0.507
	Yes	6 (8.3%)	34 (12%)	
Morning stiffness	No	15 (20.8%)	73 (25.7%)	0.482
	Yes	57 (79.2%)	211 (74.3%)	
ESR	< 20	23 (31.9%)	112 (39.4%)	0.301
	≥20	49 (68.1%)	172 (60.6%)	
CRP	< 10	32 (44.4%)	115 (40.5%)	0.635
	≥10	40 (55.6%)	169 (59.5%)	
WBC	Mean ± SD	8.1 ± 1.9	8.4 ± 2.0	0.293
RBC	Mean ± SD	4.9 ± 0.7	5.0 ± 0.6	0.580
Anemia	No	46 (63.9%)	180 (63.4%)	1.000
	Yes	26 (36.1%)	104 (36.6%)	
SJ	Fused	30 (41.7%)	103 (36.3%)	0.478
	Unfused	42 (58.3%)	181 (63.7%)	
BH	Mean ± SD	2.3 ± 1.3	2.2 ± 1.3	0.555

BASFI, Bath Ankylosing Spondylitis Functional Index; BASDAI, Bath Ankylosing Spondylitis Disease Activity Index; BMI, body mass index; PGA, Patient Global Assessment of Disease Activity; ESR, erythrocyte sedimentation rate; CRP, C-reactive protein; SJ, sacroiliac joint; BH, Bath Ankylosing Spondylitis Radiology Index-Hip.

### Internal evaluation of the ensemble DL models

The ensemble DL models were assessed using 5 fold cross-validation. First, the training set images were divided into five non-overlapping complementary subsets. Four subsets were used for each training round to train the model, while the one remaining subset was utilized for validation. This procedure was performed five times. Thus, a reliable validation approach was used to express the outcomes of the five validations as mean ± standard deviation.

**Figure 1:**
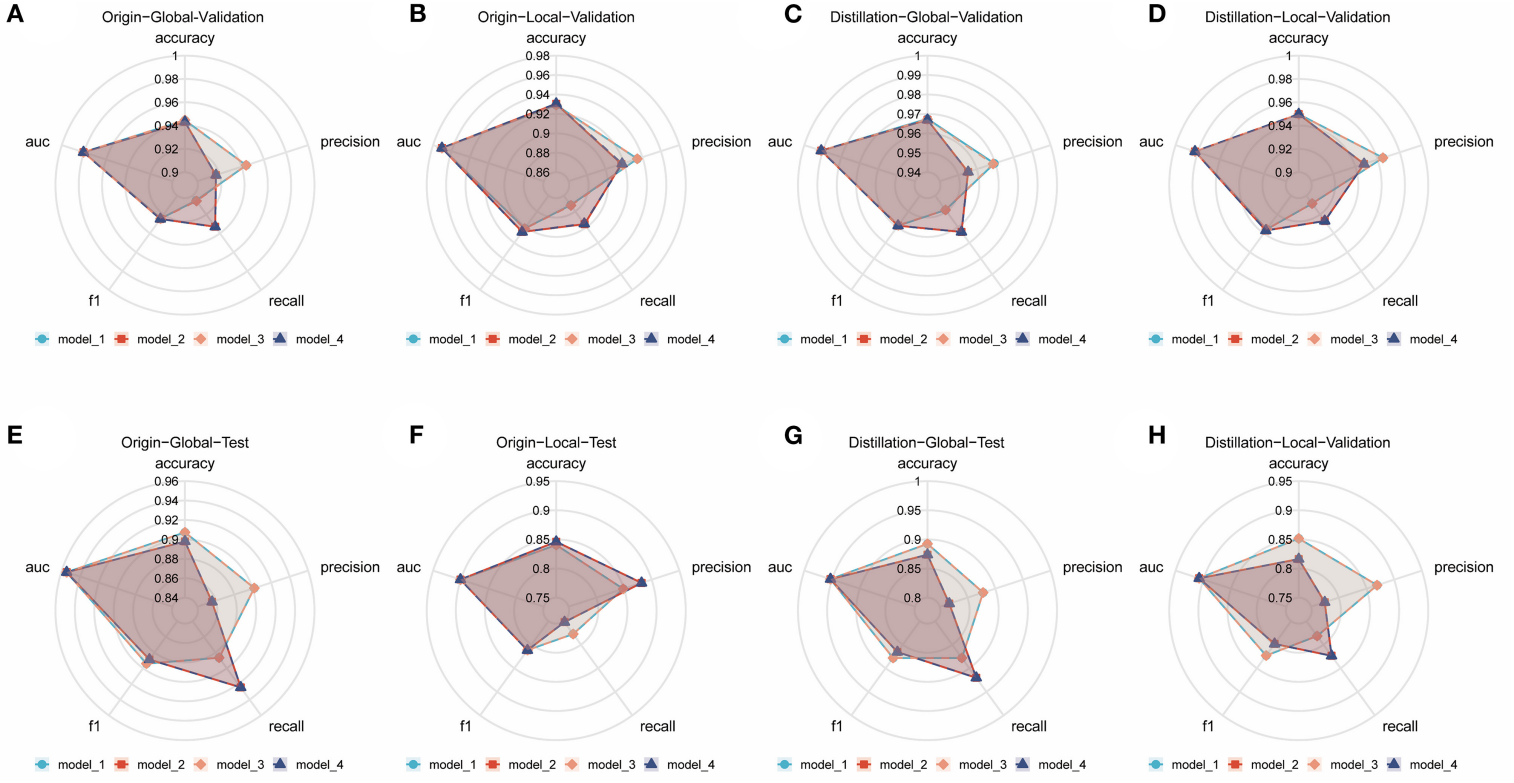
Comparison of different ensemble DL models in each internal validation dataset and external test dataset. **(A)** Comparative radar plot of different ensemble DL models in the origin-global internal validation dataset. **(B)** Comparative radar plot of different ensemble DL models in the origin-local internal validation dataset. **(C)** Comparative radar plot of different ensemble DL models in the distillation-global internal validation dataset. **(D)** Comparative radar plot of different ensemble DL models in the distillation-local internal validation dataset. **(E–H)** show the comparison of the performance of the models validated in the above dataset in the external test dataset (Model 1, model 2, model 3, and model 4 represent the models with the top 5 AUCs in each dataset using different methods of integration). Model 1: the prediction results of the 5 sub-models are directly averaged with a classification threshold of 0.5. Model 2: the prediction results of the five sub-models are directly averaged with a classification threshold of the optimal cut-off value in the training set. Model 3: the prediction results of the 5 sub-models are weighted and averaged with a classification threshold of 0.5. Model 4: the prediction results of the 5 sub-models are weighted and averaged with a classification threshold of the optimal cut-off value in the training set.

Numerous comparison tests were conducted to develop a better ensemble DL model. First, the training set was distilled, that is, the training set samples were trimmed, and the images with higher quality were retained; the quality of the images was assessed by two radiologists to examine the effect of the quality and quantity of training samples on the model. Finally, a training set with a sample size of 3,905 was acquired. Second, the images were cropped to examine whether the model—in the datasets before and after distillation—performs better in diagnosis when trained using only the local image of the sacroiliac joint or the global image of the whole pelvis. The best ensemble DL model was selected for each of the four datasets by using the aforementioned method. Information about the DL model training and comparison is presented in Figures 1A–D, which displays the overall performance of four ensemble DL models.

### External evaluation of the ensemble DL models

**Figure 2:**
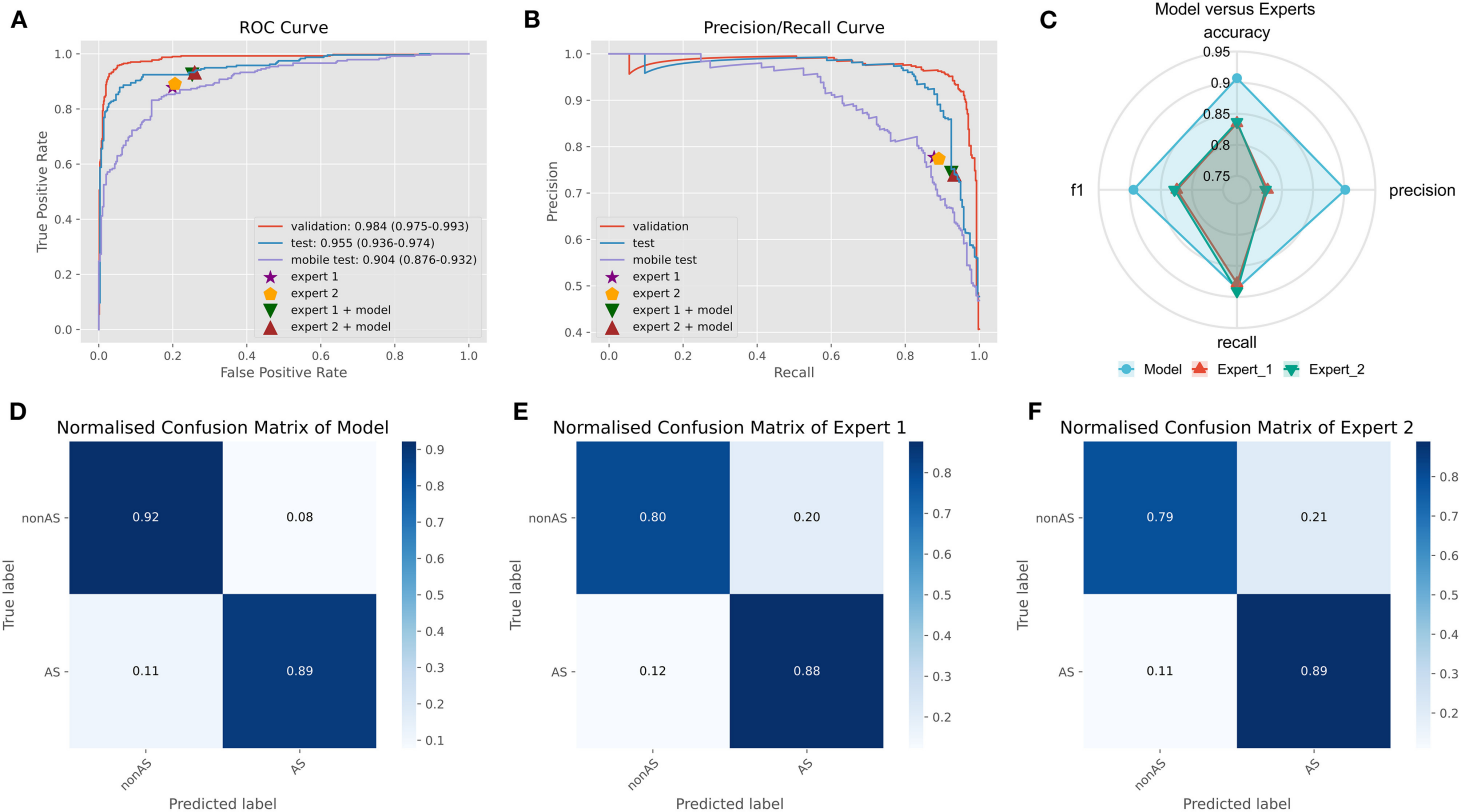
Comparison of ensemble DL model and human experts. **(A)** The ROC curve of ensemble DL model in the internal validation dataset, external test dataset, and smartphone-captured images dataset. The performance of two experts with and without model assistance in the external test dataset. **(B)** The PR curve of ensemble DL model in the internal validation dataset, external test dataset, and smartphone-captured images dataset. The performance of two experts with and without model assistance in the external test dataset. **(C)** Comparative radar plot of ensemble DL model and human experts in the external test dataset. **(D)** Normalized confusion matrix of ensemble DL model in the external test dataset. **(E)** Normalized confusion matrix of expert 1 in the external test dataset. **(F)** Normalized confusion matrix of expert 2 in the external test dataset.

On an external test set made up of 539 cases from three centers, the four best ensemble DL models established above were studied. The results are displayed in Figure 2, which also includes the results of the two experts' diagnoses. Table 3 presents the metrics employed for evaluating the model and experts' diagnosis.

**Table 3:** Comparison of ensemble DL model and human experts.

**Metrics**	**TP**	**TN**	**FP**	**FN**	**Accuracy**	**Precision**	**Recall**	**F1**	**FPR**
Model	211	278	23	27	0.91	0.90	0.89	0.89	0.08
Expert 1	209	241	60	29	0.83	0.78	0.88	0.82	0.20
Expert 2	212	239	62	26	0.84	0.77	0.89	0.83	0.21
Expert 1 with model	220	225	76	18	0.83	0.74	0.92	0.82	0.25
Expert 2 with model	222	223	78	16	0.83	0.74	0.93	0.83	0.26

The origin-global top five ensemble model exhibited the best performance, as shown in Figures 1E–H. The final diagnosis was determined by merging the model with the diagnostic judgment of the expert, and this result was contrasted with the outcome of utilizing the model alone or having a single expert read the images alone. The recall of the diagnostic increased further with the help of the model. This will further lower the rate of underdiagnosis.

**Figure 3:**
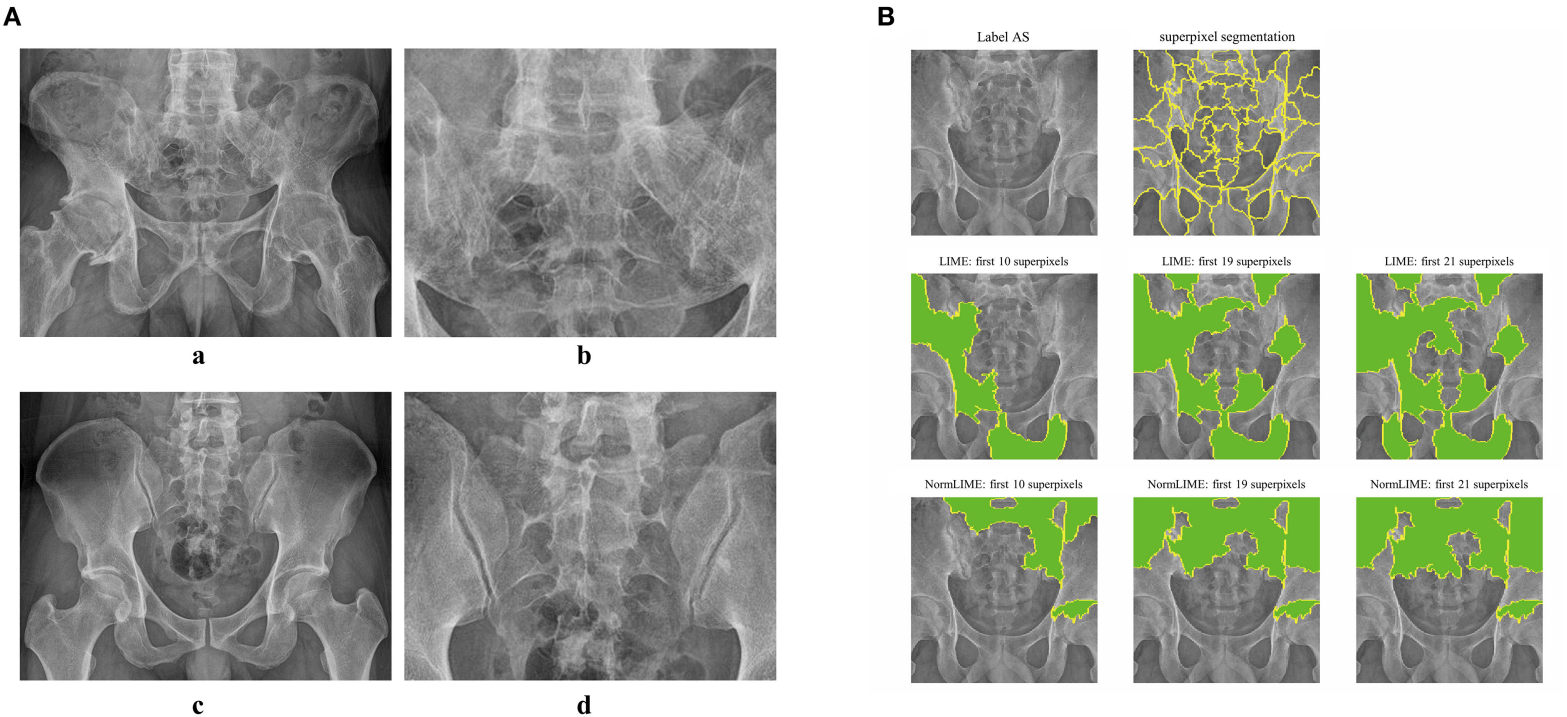
Example images and interpretative analysis of the ensemble DL model. [**(A)** a, b] Example image from a patient with AS. [**(A)** c, d] Example image from patient without AS. **(B)** LIME and NormLIME techniques to visualize the focused areas of interest of the submodel of ensemble DL model.

Because DL models are “black boxes,” assessing the veracity of their conclusions is challenging. As a result, we used the LIME and NORMLIME techniques to illustrate the key characteristics of an image. The image was divided into superpixel blocks by using the LIME and NORMLIME techniques, and these superpixel blocks were then sorted. The LIME and NORMLIME map of one submodel of the ensemble DL model is illustrated in Figure 3. The green areas are the areas that the model focuses on. The first 19 superpixels in LIME and NORMLIME show that the model reasoned mainly through the sacroiliac joint and other parts of the pelvis bone, which are generally consistent with the features that experts focus on. This demonstrates the reliability of the model inference.

### Performance of the ensemble model on smartphone-captured images

It is cumbersome to immediately upload the image to the model for prediction because X-ray equipment in China is typically not connected to the Internet. Using a smartphone to capture a photograph and transmit it to the model is the simplest solution to this issue. This method results in some image quality loss when compared to the original but does not greatly affect the DL model's diagnostic performance.

We used a smartphone to capture a photograph of each PXR in the external validation set for validation to validate the model's robustness. During the shooting procedure, we strived to preserve the original image's details. In smartphones, images are stored in JPEG format. The model was trained on clear original images; however, the results demonstrate that images captured using smartphones also provide acceptable results (AUC: 0.904, CI: 0.876–0.932) (Figure 2A).

### Evaluation of clinical prediction model

**Figure 4:**
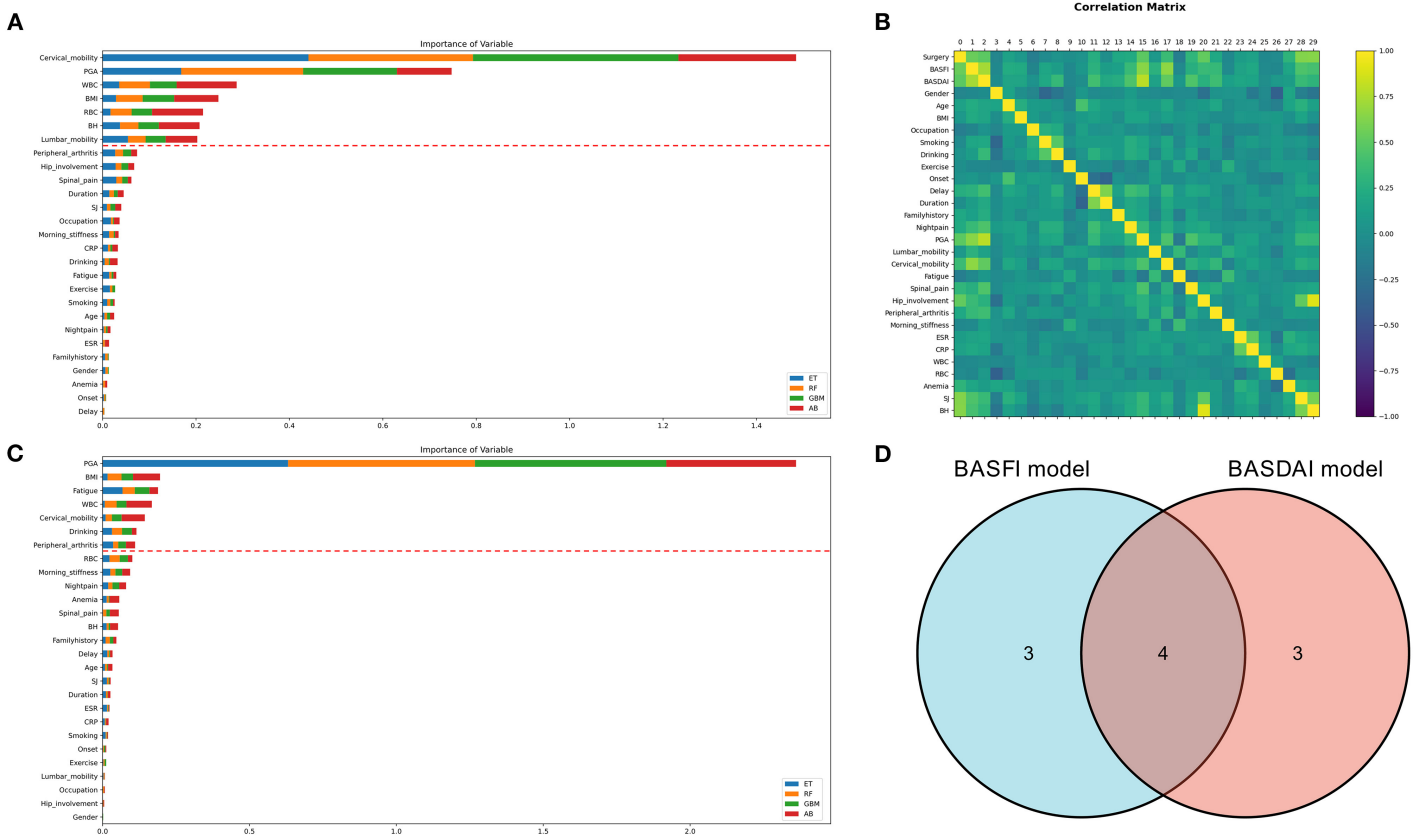
Variable selection for clinical prediction models. **(A, C)** The variable importance ranking for constructing the BASFI prediction model and BASDAI prediction model, respectively. **(B)** Correlation heat map of the variables in the training set. **(D)** Venn diagrams for the above two models incorporating variables (ET, Extra Tree Regressor; RF, Random Forest Regressor; GBM, Gradient Boosting Machine Regressor; AB, AdaBoost Regressor).

Two clinical prediction models were constructed using the clinical data of patients with AS. The correlation between the variables is depicted in Figure 4B. ML techniques were employed to only incorporate important factors in the modeling process due to the duplicated information of variables. The evaluation of each variable's importance performed using four ML algorithms were combined, and the top seven variables were selected as the most crucial for constructing the prediction models. Finally, two prediction models have been constructed: the BASDAI prediction model and the BASFI prediction model. The two models' variables are shown in Figure 4. The variables used in both models are cervical mobility, PGA, WBC, and BMI.

**Figure 5:**
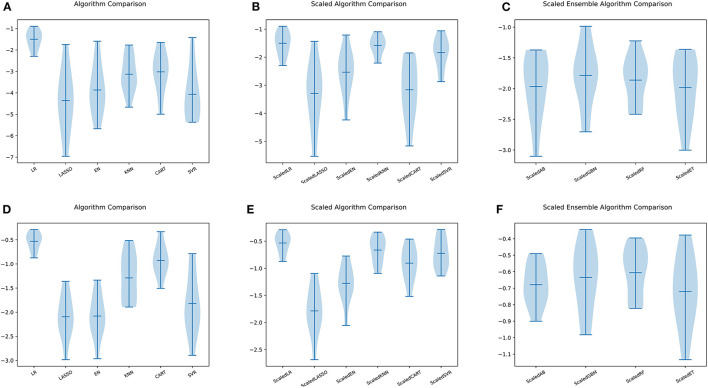
Comparison of ML models. **(A)** Six ML algorithms for BASFI prediction before standardization. **(B)** Six ML algorithms previously mentioned for BASFI prediction after standardization. **(C)** Four ensemble ML algorithms for BASFI prediction after standardization. **(D)** Six ML algorithms for BASDAI prediction before standardization. **(E)** Six ML algorithms previously mentioned for BASDAI prediction after standardization. **(F)** Four ensemble ML algorithms for BASDAI prediction after standardization (LR, Linear Regressor; LASSO, Least Absolute Shrinkage and Selection Operator; EN, Elastic Net; KNN, K Nearest Neighbors; CART, Classification and Regression Trees; SVR, Support Vector Regression; AB, AdaBoost Regressor; GBM, Gradient Boosting Machine Regressor; RF, Random Forest Regressor; ET, Extra Tree Regressor).

**Figure 6:**
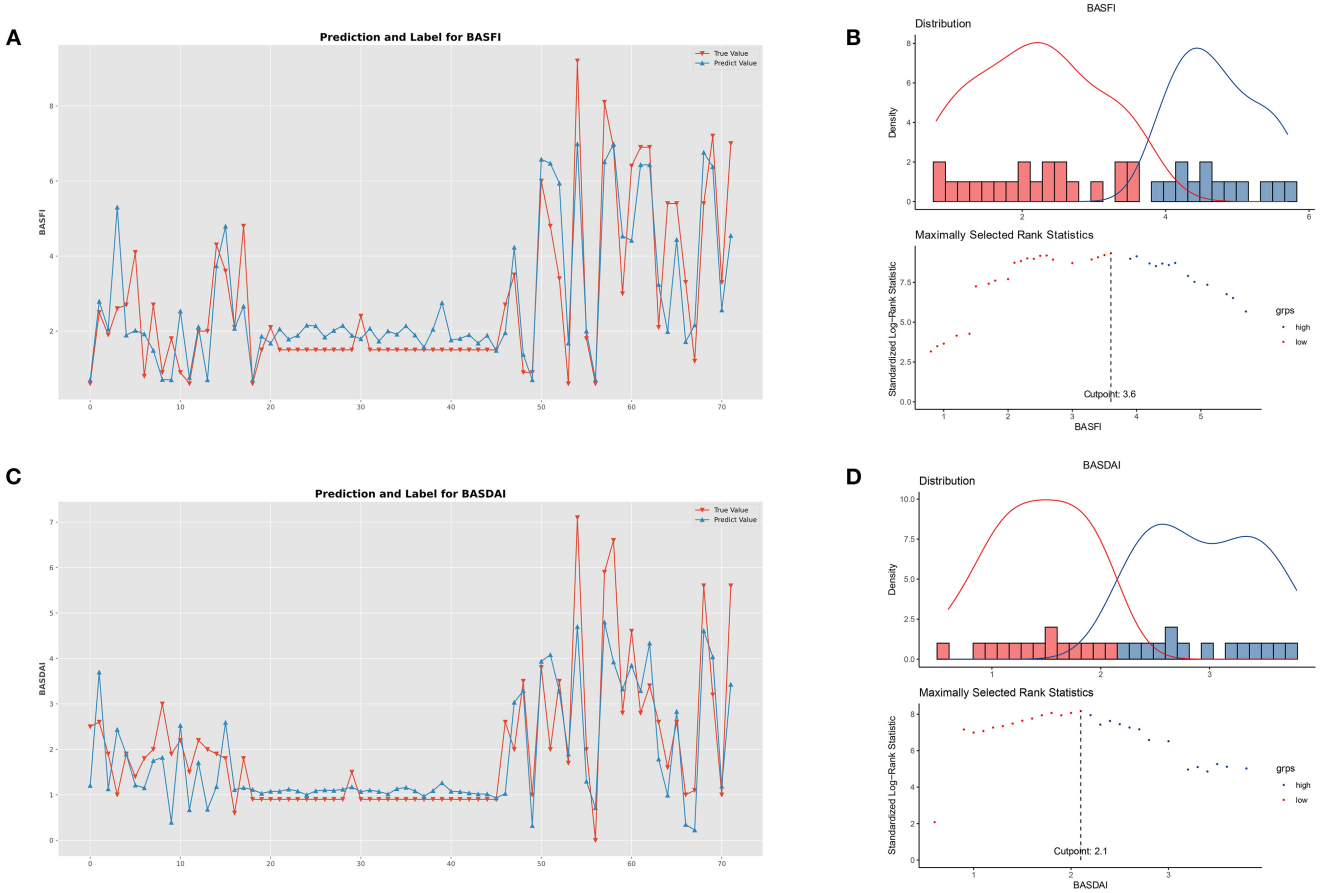
Performance of clinical prediction models and optimal cut-off value for BASFI and BASDAI. **(A)** Predicted value and the true value of BASFI. **(B)** Distribution and an optimal cut-off value of BASFI. **(C)** Predicted value and the true value of BASDAI. **(D)** Distribution and an optimal cut-off value of BASDAI.

The models' performance in the training set was assessed using 10 fold cross-validation, and the assessment measured mean square error (MSE). The data were standardized (22), and 10 ML models were trained for comparison (Figure 5). The parameters of the models were then further optimized using a grid search optimization technique (23, 24). The performance of the models was further enhanced by applying the stacking approach in ensemble learning to combine the final set of four best-performing models into the final ensemble ML model. The correlation between the model's true and predicted values in the external test set was then depicted in Figure 6.

**Figure 7:**
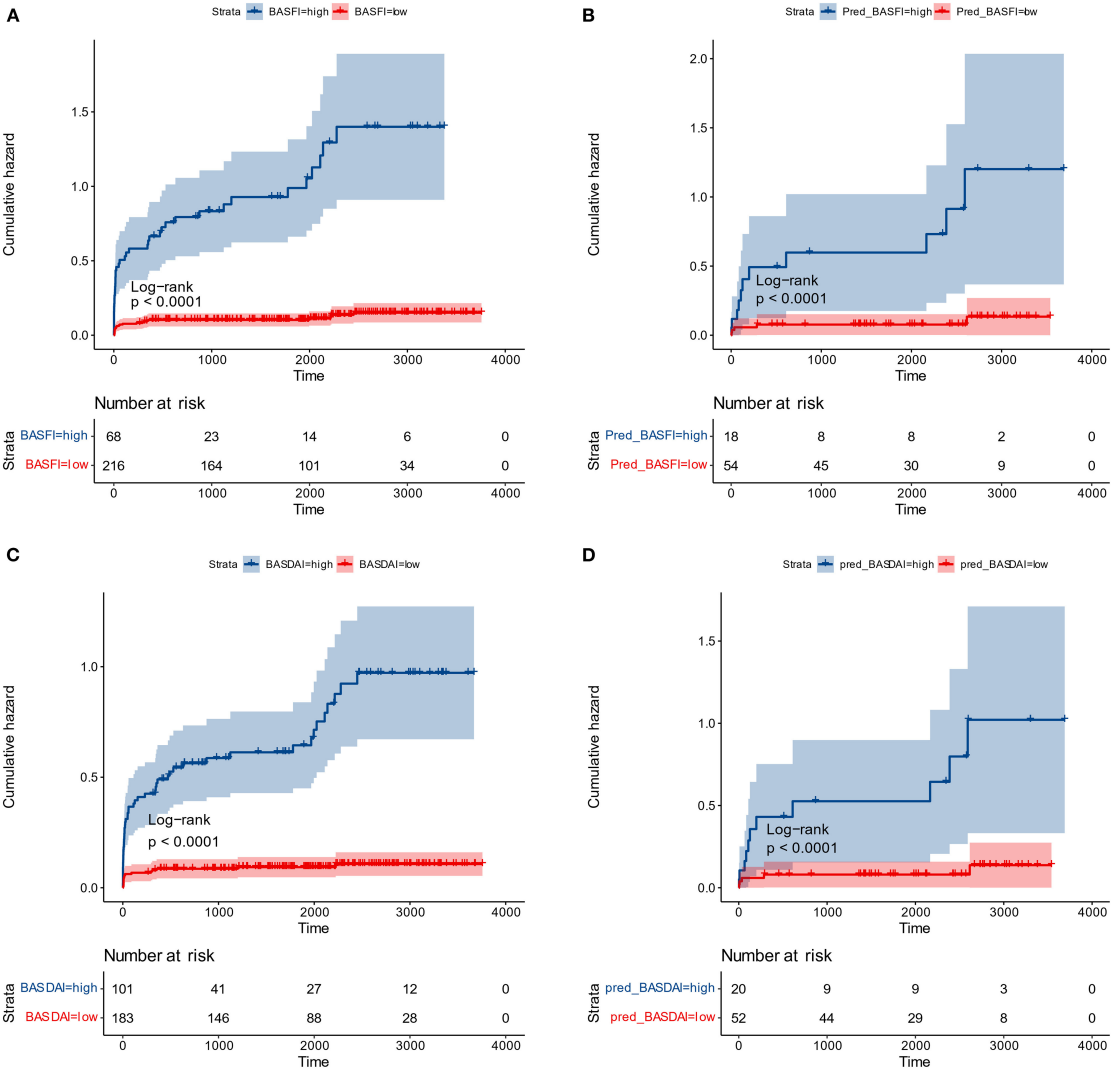
Cumulative surgical risk curves. **(A)** Cumulative surgical risk curve for BASFI in the training dataset. **(B)** Cumulative surgical risk curve for BASFI in the test dataset. **(C)** Cumulative surgical risk curve for BASDAI in the training dataset. **(D)** Cumulative surgical risk curve for BASDAI in the test dataset.

The optimal cut-off values for BASFI and BASDAI were 3.6 and 2.1, respectively. According to the optimal cut-off value, patients were divided into two subgroups; the cumulative surgical risk curves with *p*-value < 0.05 are shown in Figure 7, revealing a substantial difference in the clinical trajectory and outcome between the two subgroups.

Following the same cut-off values, patients from the external test set were pooled, and cumulative surgical risk curves were drawn (Figure 7). The model effectively differentiated patients between two subgroups with different clinical trajectories, as indicated by log-rank analysis with *p*-value < 0.05. We identified the high-risk group as those patients who were more likely to require their first surgery in a shorter period of time. If the first receiving institution lacks experience in treating AS, this group should be recommended to seek more specialized care at a more specialized facility. The prognosis for the opposing segment, the low-risk group, maybe better, but they would still require essential care. According to their respective optimal cut-off values for patient subgroups, the two models' kappa value was 0.857, demonstrating that the results of the two models are in excellent agreement and that various models can be employed for inference in real-world clinical settings based on the information about the patients.

### Model deployment

**Figure 8:**
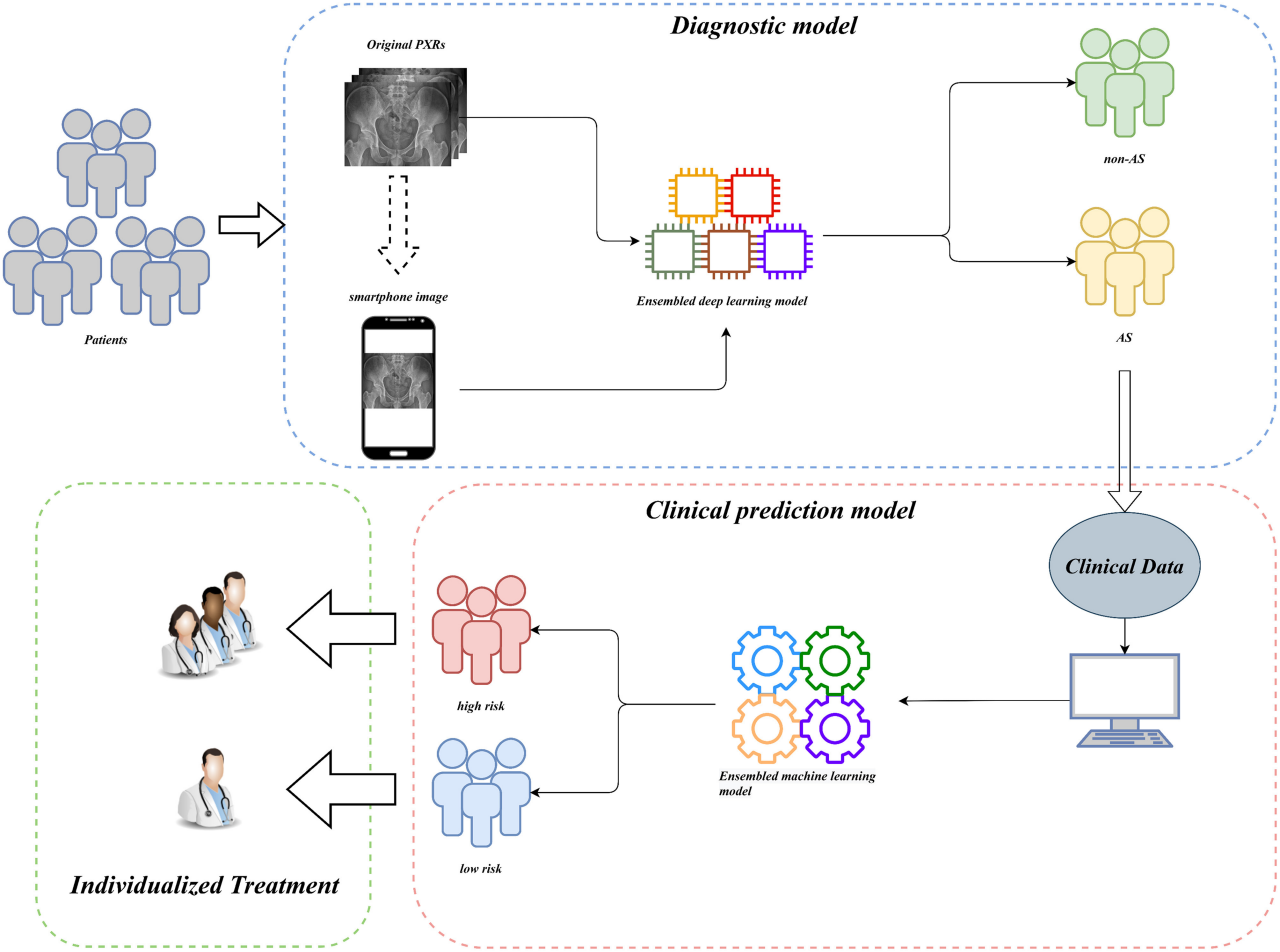
Workflow diagram of our AI system. Our AI-assisted system enables a one-stop service for diagnosis and triage.

**Figure 9:**
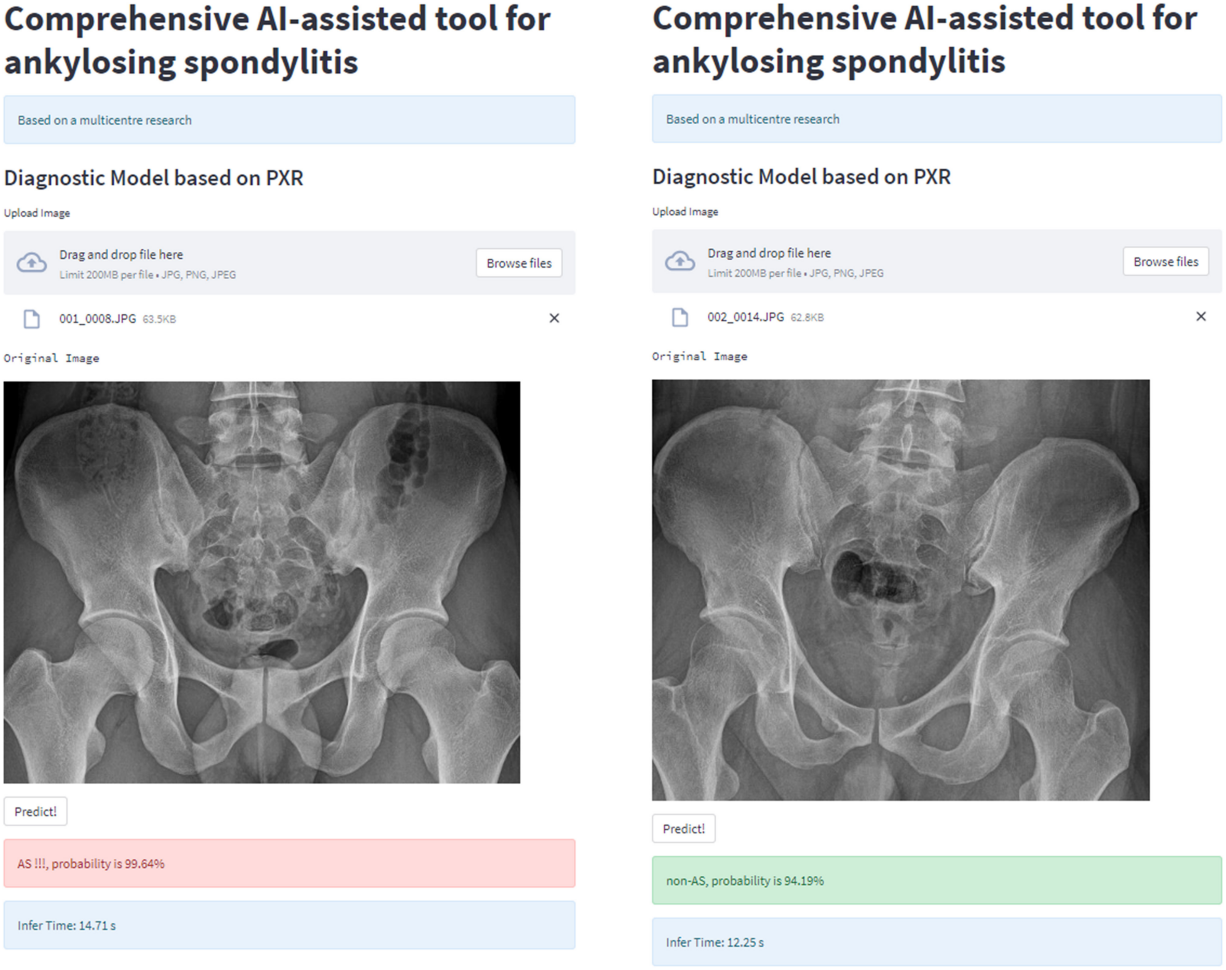
AI-assisted system examples.

For clinical usability, we deployed the model on a webpage by using Python's “streamlit” library. This makes it simple to use on both PCs and smartphones. For a quick description of the procedure and some examples, see Figures 8, 9.

## Discussion

Ankylosing Spondylitis (AS) is a debilitating and costly disease that affects both individuals and society as a whole. Early diagnosis and tailored treatment are essential for the successful management of AS. Magnetic Resonance Imaging (MRI) is an excellent method for early diagnosis of AS. Unfortunately, due to limited resources in some rural and less developed areas, MRI examinations are not always available. To address this issue, there is an urgent need for a more accessible and practical method for the early detection of AS.

In this multicenter investigation, a state-of-the-art ensemble DL model was trained and validated for the diagnosis of AS based on PXRs. Our ensemble process is straightforward and efficient. We average the prediction probabilities of the top five CNN models that performed best during internal validation to obtain the output of the ensemble model for final inference. These five CNN models are ResNet50_vd_ssld, ResNet101_vd_ssld, MobileNetV3_large, DenseNet201, and DarkNet53 (see methods Section for more information on the models). The ensemble DL model demonstrated strong performance in the internal validation set, yielding precision, recall, and AUC values of 0.94, 0.91, and 0.98, respectively. In addition, the model was validated in the multicenter external validation set, yielding precision, recall, and AUC values of 0.90, 0.89, and 0.96, respectively. In the external validation set, its diagnostic performance outperformed that of two human experts. Interestingly, recall improved further when human expert diagnoses were combined with model diagnoses. Higher recall can lessen missed diagnosis, which is better for people with clinically suspected AS. Thus, not only can the proposed ensemble DL model assist primary hospitals without experts in enhancing the accuracy of AS diagnosis but can also further improve the diagnostic performance of seasoned experts at tertiary hospitals.

It is difficult to directly input images from X-ray equipment into our model for inference in actual clinical scenarios in China because X-ray equipment is typically not connected to the Internet. The simplest solution to this issue is to use smartphones to capture photographs, which are subsequently inputted into the model for inference. Therefore, to deploy the model on the website—which can be utilized on the PC side and can deliver photographs directly into the model on the smartphone side—we used Python's “streamlit” library. Anyone can quickly and easily start using the model, thanks to its user-friendly interface, providing hospitals in rural and underdeveloped areas access to AI diagnostics. Compared with images obtained using X-ray equipment, images captured using smartphones suffer an unavoidable loss of quality. However, the model operates quite robustly on images captured using smartphones by validating the inference performance of smartphone-captured images by using a multicenter external test set of smartphone-captured images. The AUC value of the model is 0.904, which is comparable to the results obtained from the original images by human experts. Thus, especially for inexperienced radiologists in underdeveloped areas, this AI diagnostic tool based on smartphone-captured images can considerably increase the effectiveness of AS diagnosis.

DL models have long been criticized for being “black boxes,” where the output is good, but nothing is known about the inference process, and the findings are uninterpretable. The benefit of using CNNs is that the focus of the model's attention can be visualized, reducing the neural network's “black box” status. Understanding the point of focus of a model's inference is crucial for developing the model and improving its level of effectiveness. It also aids in increasing the confidence in the model's inference. In this study, we used the LIME and NORMLIME techniques to visualize the inference process of the model. LIME relies on a local interpretation that is specific to the current sample, whereas NORMLIME utilizes a global interpretation that uses a certain number of samples and enables some noise reduction. LIME and NORMLIME techniques are interpretative algorithms that divide an image into superpixels, calculate and rank each superpixel's weight, and then identify the blocks of superpixels that are the focus of the model and play the most significant part in the inference process. As shown in Figure 3, the submodel of the ensemble model focuses on the condition of the sacroiliac. This also illustrates the reliability of the model output because it is consistent with the attention of human experts. In addition to the aspects that human experts concentrate on, the model concentrates on several additional features, such as the areas of the sacrum, iliacus, situs, and hip joints, which may be affected by image noise or may represent prospective new features in the PXR. Further research on these novel imaging features is urgently required.

DL models need to be trained using large-scale datasets to achieve excellent performance, and CNNs are no exception. However, because AS is an uncommon disease, obtaining a large-scale PXR dataset is challenging. It is challenging to prevent underfitting and overfitting of the model with an insufficient sample size (25, 26). This affects the model's generalization performance. The most common problem encountered during CNN training is overfitting. In this study, we employed several effective model training strategies such as data augmentation (27), transfer learning (28, 29), and ensemble learning (20, 30). To explore the optimal ensemble DL model, based on the aforementioned strategies, we conducted numerous comparison experiments, such as training different architectures of CNN networks, training using datasets of different sizes, and training using global and local images. The 5 fold cross-validation results revealed that the ensemble model constructed using the top five models developed using the origin-global dataset is the best option. Surprisingly, when the model was trained using the distilled dataset, the performance in the validation set improved; however, the performance in the test set decreased. This may be because not all PXRs from a multicenter are of high quality, and training the model by using only high-quality images will result in its underperformance in generalization to samples with diverse image qualities. This also demonstrates that CNN training requires a large dataset with diversity rather than a refined dataset with a small number of samples. Furthermore, we found that although the results of the models trained using different datasets differed, their final training results were quite satisfactory. This suggests that our model training strategies are quite robust and can be applied to similar studies in other medical fields.

CNNs have been successfully employed for enhancing diagnostic performance in a variety of medical imaging datasets, such as the identification of aberrant electrocardiograms (19), early diagnosis of biliary atresia (20), and detection of pelvic injuries (21). Robust diagnostic models have been developed that, when used in clinical scenarios, will drastically lower the rate of misdiagnosis. These models are more effective than human experts. Unfortunately, the greater diagnosis efficiency alone may not have a noticeable effect on the patient's prognosis. Doctors in developing nations and underdeveloped regions may lack advanced diagnostic abilities. In addition, they could be less informed about the disease's severity and clinical trajectory; this would lead to patients having a proper diagnosis but failing to receive prompt and efficient treatment, which is identical to a misdiagnosis or missed diagnosis. Therefore, in this study, two clinical prediction models for AS were developed to predict the clinical trajectory of the disease. To enhance the performance of the models with a relatively small sample size, an ensemble learning technique was also adopted for creating the clinical prediction models. Two clinical prediction models, namely BASFI prediction model and BASDAI prediction model, were constructed to classify patients into high-and low-risk groups based on their optimal cut-off values. The high-risk group is defined as patients who were more likely to require surgical treatment for severe spinal or hip lesions in a shorter period of time. We utilized this definition of the high-risk group because in patients with AS, spine or hip pathology is the primary factor contributing to a deterioration in the quality of life, and surgery is ultimately necessary when conservative therapy is ineffective at alleviating symptoms. Therefore, the point in time when surgery is required is an excellent indicator of a patient's condition. Our clinical prediction models enable early detection of the high-risk group and patient triage *via* this node and enable individualized AS therapy. Furthermore, the most logical distribution of healthcare resources can be maximized by advising patients in the high-risk group to go to specialized hospitals for specialized and individualized treatment while providing the required treatments for patients in the low-risk group.

In summary, we developed a comprehensive AI-assisted system that combines AS diagnosis and clinical prediction. The proposed ensemble DL model, which is presently the best ensemble DL model in this field, is based on PXR images and has precision, recall, and AUC values of 0.90, 0.89, and 0.96, respectively, on a multicenter external validation set. The proposed clinical prediction model can stratify patients with confirmed AS, identify high-risk patients among them, and aid in bringing them to greater medical attention. This has important ramifications for encouraging individualized AS therapy and enabling sensible resource allocation in the healthcare system.

## Methods

### Patients and data collection

The Ethics Committee of the First Affiliated Hospital of Guangxi Medical University authorized this multicenter study, and each participant filled out a written consent form.

**Figure 10:**
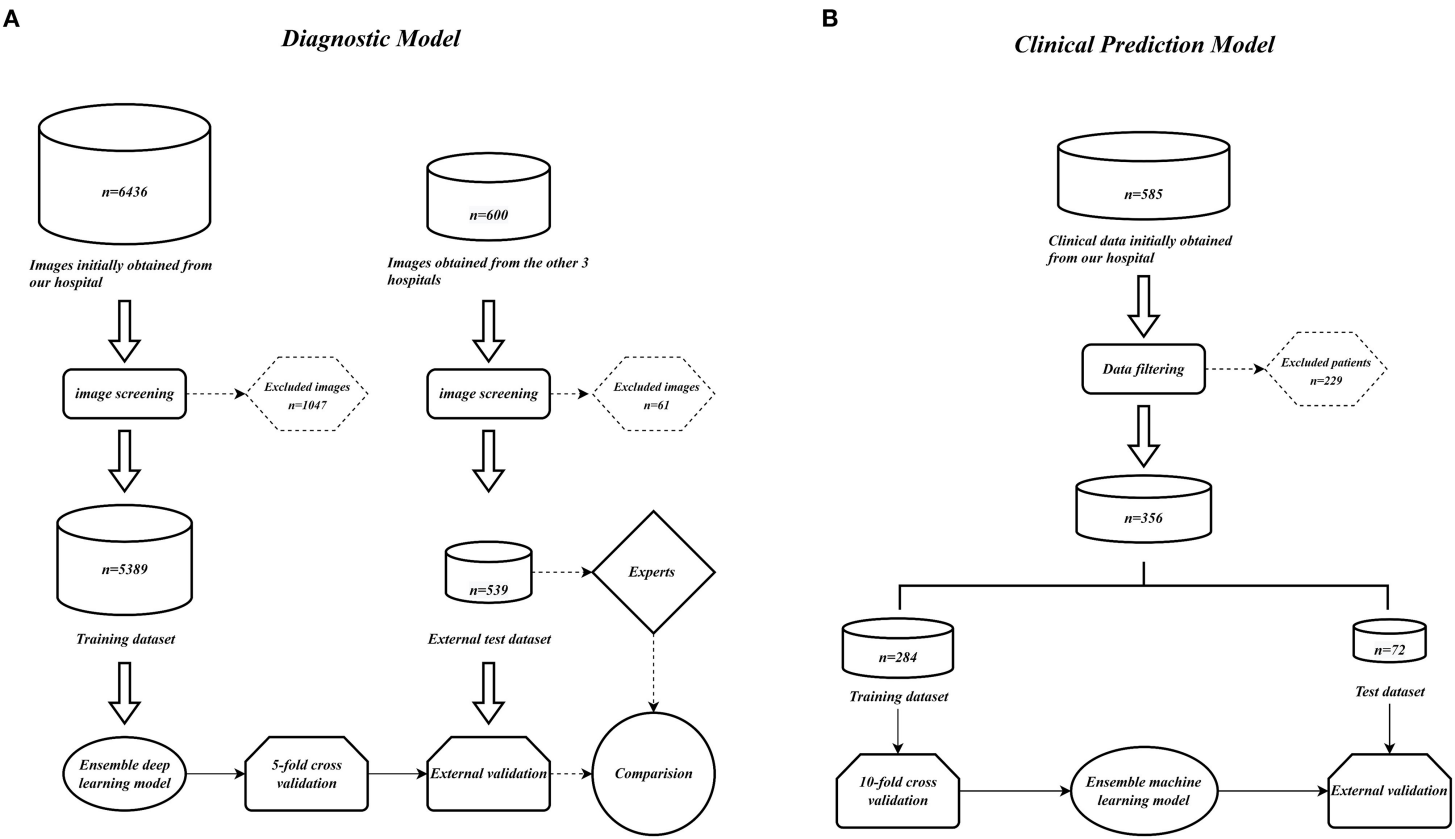
Model construction flow charts. **(A)** Ensemble DL model construction flow chart. **(B)** Ensemble ML models construction flow chart.

We collected 6,436 PXRs by using the electronic imaging system of the First Affiliated Hospital of Guangxi Medical University between March 2014 and April 2022. From three additional centers' electronic imaging systems, 600 PXRs were collected (Three additional centers: the First People's Hospital of Nanning, Wuzhou Red Cross Hospital, People's Hospital of Baise). Patients who satisfied the diagnostic standards of the modified New York criteria (3) were included in the AS group, and the PXRs of patients with a diagnosis of non-AS were used as controls. Two radiologists with more than 10 years of PXR reading expertise assessed the initially included PXR images to weed out those that had an inadequate imaging quality. Finally, 2,015 PXRs from the AS group and 3,374 PXRs from the non-AS group from our hospital were used. A total of 238 PXRs in the AS group and 301 PXRs in the non-AS group from other centers were included. The data collecting process is depicted in Figure 10A.

We retrospectively gathered the data, including general information, epidemiological indicators, clinical indicators, laboratory results, and imaging scores, of 585 patients with confirmed AS in our hospital by using the electronic case system and electronic imaging system to construct a clinical prediction model for AS. Comprehensive data were available for 356 patients. The aforementioned patients were randomly divided into a training set and a test set in the ratio of 7:3. Table 2 lists the information regarding the patients in the training and test sets, and the data collection flowchart is displayed in Figure 10B.

### Subgroups of training set images

To assess the robustness of the proposed ensemble DL model and determine the optimal model, the training set was processed further. First, the training set samples were distilled; a portion of the images with lower image quality was removed, and the images with higher image quality were retained and used to construct the new training set. Two radiologists with 10 years of PXR reading expertise evaluated the image quality. Next, a new training set with a sample size of 3,905 was obtained, with 1,572 cases in the AS group and 2,333 cases in the non-AS group. Images with bounds in four directions just slightly beyond the sacroiliac joint were used for training to compare the effects of local images of the sacroiliac joint vs. global images of PXR on the training of the model. Finally, four training sets were obtained: global image training set before distillation, local image training set before distillation, global image training set after distillation, and local image training set after distillation.

### Ensemble DL model training and internal validation

We constructed an AI-assisted model for AS diagnosis by using deep CNN and ensemble learning. First, single CNN models were developed in the training set, and a total of ten distinct CNN models were trained: ResNet50, ResNet50_vd_ssld, MobileNetV3_small_ssld, DenseNet201, ResNet101_vd_ssld, MobileNetV3_large, MobileNetV3_large_ssld, MobileNetV3_small, DarkNet53, and Xception41. For more information on the models discussed in our search results, please refer to the PaddleX Model Zoo at https://paddlex.readthedocs.io/zh_CN/release-1.3/appendix/model_zoo.html. This page contains a comprehensive list of the various models available, along with detailed descriptions, usage examples, and performance metrics.

Before entering the CNN network, we preprocessed the images as follows to implement data augmentation in order to increase the robustness of the model training: 1. Resize the image to a new image with a short edge length of 512 pixels, according to the original scale; 2. Randomly crop a region of 224 pixels in width and height on the new image; 3. Perform random rotation, random horizontal inversion, random vertical inversion, and normalization operations on the crop region. However, for the validation and test sets, we removed the above image augmentation operations and simply resized the original image into a new image with a short edge of 256 pixels at the original scale, cropped the center region of the new image with a width and height of 224 pixels, and performed the normalization operation afterwards.

We employed a 5 fold cross-validation approach for internal validation throughout model training to assess the resilience of the process. The training set was randomly divided into five equal-sized, non-overlapping subsets. Four of these subsets were used to train the model during each training session, while the fifth subset was used to validate the model. The above process was repeated five times; the metrics used for validation included accuracy, precision, recall, AUC, and F1 score. In addition, transfer learning (28, 29) and data augmentation (27) were used to increase the accuracy of the models. All the models were pretrained on ImageNet-1 k before being trained on our training set, and the training samples were randomly rotated, cropped, horizontally flipped, and vertically flipped. The training and validation sets of images underwent standardization. The top five models in terms of AUC during internal validation were then used to create an ensemble DL model by using an ensemble learning technique. We employed two ensemble approaches. One included calculating the final prediction probability by averaging the output prediction probabilities of the five models. The other approach involved assigning each of the five models a distinct weight based on their AUC values, averaging the output probabilities with the weights, and using the weighted average as the final prediction probabilities. Four training sets were used in this study, and all the models were trained using the aforementioned method. For each training set, the best model was selected, and the four models were compared to determine the best one.

### External validation of ensemble DL models and comparison with human experts

We used 539 PXRs from three additional clinical centers as an external test set for multicenter external validation of the proposed ensemble DL model for assessing the model's generalization ability. The evaluation metrics included accuracy, precision, recall, AUC, and F1 score. We utilized the diagnostic outcomes of human experts as a benchmark to more accurately assess the model's performance. Without seeing any patient's PXR beforehand and without having access to the patients' basic information or clinical data, two radiologists with more than 10 years of expertise in interpreting pelvic X-rays reviewed 539 PXRs. Then, the accuracy, precision, recall, AUC, and F1 scores of the ensemble DL models and the human expert were compared. Next, to assess the performance of the human expert with the assistance of the model, we combined the results of the human expert and the model's evaluation. The method is as follows: whenever a human expert or the model judges the PXR to be AS, the final result is recorded as AS; otherwise, it is recorded as non-AS. The accuracy, precision, recall, AUC, and F1 scores were calculated separately for the two experts aided by the model.

We used the NORMLIME approach, which enhances the LIME (31) method and is an advanced way of model interpretation to visualize the inference process of each submodel in the ensemble DL model to validate the model performance. By dividing the image into superpixel blocks and observing the superpixel blocks that the model deems to be most relevant, the reliability of the model judgment was evaluated.

### Ensemble DL model validation on smartphone-captured images

To assess the model's performance on the smartphone-captured images, we snapped an image of each PXR in the external validation set by using a smartphone and assembled a test set of the resulting images. The images were captured such that as many aspects of the original image were preserved as possible. For example, the shooting angle and proximity to the computer screen were adjusted to minimize the difference between the original image displayed on the phone and the computer. The metrics evaluated for the model included accuracy, precision, recall, AUC, and F1 score. To evaluate the diagnostic efficacy of the expert in combination with the model while using smartphone-captured images, the aforementioned metrics were also examined for the expert's model-aided predictions.

### Training and validation of clinical prediction models

The 356 patients included in the study were randomly divided into a training set and a test set in the ratio of 7:3. The test set was utilized for external model validation, whereas the training set was used for model construction and training. BASFI, BASDAI, whether to undergo surgery and time to first surgery were not included in the model construction as these are variables for assessing the outcome. Four ensemble models based on decision tree models were used to compute the importance of each variable. The importance determined from the four models was then added to determine the importance of each variable. We selected the top seven important variables for modeling and used them for training six single ML models before they were standardized. Following standardization, the variables were used for training the six models as previously mentioned. The variables following standardization were then used to train four ensemble ML models. All six models were trained using 10 fold cross-validation to ensure the robustness of the model evaluation outcomes. We constructed two prediction models: the BASFI prediction model and the BASDAI prediction model. The grid search technique was used to select the hyperparameters of the models. The MSE for each model was compared. To create the final ensemble ML models, the top four models were selected and combined using the stacking approach. The test set was used to evaluate the ensemble models, and the MSE values were computed.

### Identification of high-risk groups among patients with AS

In accordance with the survival analysis approach, we identified the first surgical procedure as the end event and the interval between the patient's initial visit and the first surgical procedure as the time without surgery. By finding the best difference between the data on each side of a point in the training set, we obtained the optimal cut-off values for BASFI and BASDAI by using log-rank tests. The R packages “survival” and “suivminer” were used to complete this operation. For BASFI and BASDAI, the optimal cut-off values in the training set were 3.6 and 2.1, respectively. According to the ideal cut-off values, the patients with AS were divided into high and low BASFI groups or high and low BASDAI groups, respectively, to investigate the cumulative surgical risk curves. Because the cumulative surgical risk for patients in the high BASFI group and high BASDAI group increased more rapidly, they were classified as a high-risk group. In the test set, patients were separated into high-and low-risk groups based on the predicted values of the two models by using the optimal cut-off values from the training set and the cumulative surgical risk curves. A *p*-value of < 0.05 for the log-rank tests for the two models indicated that the models can successfully divide the test set's patients into high-and low-risk subgroups.

### Statistical analysis

The Delong test (32) was used to compare the AUC values. Different kappa value ranges indicate various degrees of agreement. Kappa (20) values of < 0.20, 0.20 to < 0.40, 0.40 to < 0.60, 0.60 to < 0.80, and 0.80–1.00 indicate poor, moderate, fair, good, and very good agreement, respectively. A two-sided p-value of less than 0.05 was regarded as statistically significant in all statistical tests. The AS clinical prediction models were trained using the “sklearn” library in Python 3.7, and the ensemble DL models were trained using the “paddlex” library. The “streamlit” library was used for the model deployment. Multiple interpolations performed using the “mice” package of R were added to the data used to construct the clinical prediction models. To plot cumulative surgical risk curves, the “survival” and “survminer” packages of R were used [AI Studio's cloud servers are used to train DL models (https://aistudio.baidu.com). All DL models are trained on NVIDIA Tesla V100 Graphic Processing Unit (GPU)].

## Data availability statement

The code used to evaluate the models performance will publicly available. Usage of this code is for academic purposes only and the operation packages were accessed upon request. The raw data supporting the conclusions of this article will be made available by the authors, without undue reservation.

## Ethics statement

The studies involving human participants were reviewed and approved by Ethics Department of the First Affiliated Hospital of Guangxi Medical University. Written informed consent for participation was not required for this study in accordance with the national legislation and the institutional requirements.

## Author contributions

HLi, XT, CLi, and XZ designed the study. TL, JJ, JZ, SW, and LC analyzed the data. ZZ, CZ, XS, SH, JC, and TC did digital visualization. ZY, HG, YY, SL, CY, and WC collected data on clinical data. HLiu, SP, CH, YL, CLu, JH, QX, XW, CF, and BF collected data on image data. HLi wrote and revised the manuscript. CLi and XZ revised the manuscript. All co-authors participated in the laboratory operation.
